# C-reactive Protein/Albumin Ratio and Acute Kidney Injury after Radical Cystectomy among Elderly Patients: A Propensity Score-Matched Analysis

**DOI:** 10.1155/2020/8818445

**Published:** 2020-10-27

**Authors:** Jihion Yu, Jun-Young Park, Seungsoo Ha, Jai-Hyun Hwang, Young-Kug Kim

**Affiliations:** Department of Anesthesiology and Pain Medicine, Asan Medical Center, University of Ulsan College of Medicine, Seoul, Republic of Korea

## Abstract

**Background:**

The C-reactive protein (CRP)/albumin ratio is a useful index used to represent patient inflammation and nutritional status. Elderly patients are at the highest risk for acute kidney injury (AKI). We clarified the impact of the preoperative CRP/albumin ratio on AKI and evaluated the impact of postoperative AKI on end-stage renal disease (ESRD) among elderly cystectomy patients.

**Methods:**

We included elderly patients ≥ 65 years of age who underwent radical cystectomy. Multivariate logistic regression analysis and receiver operating characteristic curve analysis were performed to identify risk factors for AKI. Propensity score-matched analysis and conditional logistic regression analysis were performed to elucidate the impact of the CRP/albumin ratio on AKI. The incidence of ESRD was compared between the non-AKI and AKI groups at 12 months after radical cystectomy.

**Results:**

AKI occurred in 110 patients (32.2%). The CRP/albumin ratio and 6% hydroxyethyl starch amount were risk factors for postoperative AKI. The optimal cut-off value for the CRP/albumin ratio predicting AKI was 0.1. After propensity score matching, the AKI incidence in the CRP/albumin ratio ≥ 0.1 group was higher than that in the CRP/albumin ratio < 0.1 group (46.7% vs. 20.6%, *P* < 0.001), and a CRP/albumin ratio ≥ 0.1 was associated with a higher AKI incidence (odds ratio = 4.111, *P* < 0.001). The ESRD incidence was higher in the AKI group than in the non-AKI group (7.3% vs. 1.2%, *P* = 0.017).

**Conclusion:**

A CRP/albumin ratio ≥ 0.1 was associated with an increased incidence of AKI, which was associated with higher ESRD incidence among elderly cystectomy patients.

## 1. Introduction

The risk of bladder cancer increases with age [1]. Radical cystectomy is the gold standard therapy for muscle-invasive bladder cancer. However, radical cystectomy is known to be the most complicated procedure in urological surgery [2]. The rates of complications, morbidity, and mortality associated with radical cystectomy are high among elderly patients [3]. In particular, postoperative acute kidney injury (AKI) is common after radical cystectomy and is associated with increased hospital stay durations, healthcare costs, and mortality [4]. Elderly patients are at the highest risk for AKI and AKI-associated mortality [5]. Furthermore, the diagnosis of AKI among elderly patients is prone to be masked and delayed because muscle mass decreases with age [6]. Nonetheless, to date, there have been few reports about AKI after radical cystectomy for elderly patients.

C-reactive protein (CRP) is a useful marker for monitoring infection and inflammation [7]. CRP elevation is known to provide valuable short-term prognostic information for elderly patients [8]. Moreover, serum albumin is a negative acute-phase protein associated with acute inflammation, and it is also an indicator of nutritional status [9, 10]. Therefore, the ratio of CRP to albumin (CRP/albumin ratio) is used to represent patient inflammation and nutritional status, and it is known as a prognostic factor in the context of malignancy and critical illness [11, 12]. However, to our knowledge, no published studies have investigated the predictive ability of the CRP/albumin ratio on AKI after radical cystectomy for elderly patients.

Therefore, we clarified the impact of the preoperative CRP/albumin ratio on AKI after radical cystectomy for elderly patients using propensity score-matched analysis and conditional logistic regression analysis. We also evaluated the impact of postoperative AKI on end-stage renal disease (ESRD) among elderly patients.

## 2. Patients and Methods

### 2.1. Patients

Ethical approval for this study (approval no. 2019-1627) was provided by the institutional review board of Asan Medical Center, Seoul, Republic of Korea, on 18 December 2019. We retrospectively recruited and reviewed patients ≥ 65 years of age who underwent radical cystectomy, between August 2007 and December 2018, for bladder cancer treatment at Asan Medical Center. Patients with incomplete medical records and known ESRD were excluded. The requirement for written informed consent was waived by the institutional review board due to the retrospective study design. All methods were carried out in accordance with relevant guidelines and regulations.

### 2.2. Study Protocols

General anesthesia was performed according to our standard protocol [13]. General anesthesia was induced with propofol or thiopental sodium and rocuronium and maintained with sevoflurane. Arterial catheterization and central venous catheterization were performed. Tidal volume was adjusted to 8–10 mL per kg of ideal body weight, and respiratory rate was controlled to maintain an end-tidal carbon dioxide concentration of 35–40 cmH_2_O. The concentration of sevoflurane was modified to maintain a bispectral index of 40–60. The mean arterial blood pressure was maintained at > 65 mmHg with fluid and vasopressors/inotropics, such as ephedrine, phenylephrine, and norepinephrine. Crystalloids, such as plasma solution A or lactated Ringer's solution, and colloids, such as 6% hydroxyethyl starch or 5% albumin, were administered. Gelatins and plasma derivatives were not administered. Red blood cells were transfused when the hemoglobin concentration was < 8 g/dL. Reversal of neuromuscular blockade was performed with a neostigmine-glycopyrrolate mixture or sugammadex, as decided by the anesthesiologists. Intravenous patient-controlled analgesia with fentanyl was used to manage postoperative pain.

Radical cystectomy was performed with the standard technique at our center [14]. Standard or extended pelvic lymph node dissection was performed at the discretion of urologic surgeons. Standard pelvic lymph node dissection included the perivesical, obturator, internal iliac, external iliac, and distal common iliac lymph nodes. Extended lymph node dissection included the lymph nodes to the extent of the proximal common iliac artery, distal aorta, and inferior vena cava. Urinary diversion with an ileal conduit or ileal neobladder was subsequently performed according to the urologic surgeon's discretion. Ileal conduit urinary diversion was performed as follows: an ileal segment measuring 20 cm in length was harvested, sparing the distal 15 cm of the ileum, and the remaining ileal segment was reanastomosed. Both ureters were implanted in the conduit. The stoma was formed at the selected site on the surface of the abdomen. Ileal neobladder urinary diversion was performed as follows: depending on the type of neobladder, a specific length of the distal ileum (15–20 cm away from the ileocecal valve) was harvested to create an orthotopic continent diversion. Continuity of the remnant bowel was reestablished by ileoileostomy [15].

### 2.3. Definitions of CRP/Albumin Ratio, AKI, and ESRD

The CRP/albumin ratio was calculated by dividing the serum CRP level (mg/dL) by the serum albumin level (g/dL) [12]. Postoperative AKI was defined according to the Kidney Disease: Improving Global Outcomes (KDIGO) criteria: an increase in the serum creatinine level by 0.3 mg/dL from the baseline value within 2 postoperative days or an increase in the serum creatinine level by 50% from the baseline value within 7 postoperative days [16]. AKI stage was classified according to the KDIGO criteria: stage 1, increase in serum creatinine by ≥ 0.3 mg/dL or increase to ≥ 150–199% of the baseline value; stage 2, increase in serum creatinine to ≥ 200–299% of the baseline value; and stage 3, increase in serum creatinine to 300% of the baseline value, or serum creatinine ≥ 4.0 mg/dL, or initiation of renal replacement therapy [16]. ESRD was evaluated at 12 months after radical cystectomy and was defined by the initiation of dialysis therapy or an eGFR < 15 mL/min/1.73 m^2^ [17]. The eGFR was calculated using the Chronic Kidney Disease Epidemiology Collaboration (CKD-EPI) equation: eGFR_CKD‐EPI_ = 141 × (minimum of standardized serum creatinine [mg/dL]/*κ* or 1)^*α*^ × (maximum of standardized serum creatinine [mg/dL]/*κ* or 1)^−1.209^ × 0.993^age^ × (1.018 if female), where *κ* is 0.7 for women and 0.9 for men and *α* is −0.329 for women and −0.411 for men [18].

### 2.4. Data Collection

Patient demographic and clinical characteristics included sex, age, body mass index, American Society of Anesthesiologists Physical Status, comorbidities, smoking history, tumor stage, tumor grade, neoadjuvant chemotherapy, and preoperative laboratory tests performed within 2 weeks before radical cystectomy. Tumor stage was classified according to the 2010 American Joint Committee on Cancer tumor-node-metastasis staging system [19]. Tumor grade was classified according to the 2016 World Health Organization grading system [20]. Neoadjuvant chemotherapy was performed using one of the following regimens: gemcitabine and cisplatin; methotrexate and vinblastine; sulfate, cisplatin, and doxorubicin; or gemcitabine and carboplatin. Intraoperative data included operation duration, anesthesia duration, crystalloid and colloid amounts, red blood cell transfusion, estimated blood loss, intraoperative hypotension, reversal agent of neuromuscular blockade, and urinary diversion type. Estimated blood loss was calculated using the following equation: estimated blood loss (mL) = estimated blood volume (mL) × (preoperative hematocrit (%) − postoperative hematocrit (%)) + (transfused red blood cell in units × 213 mL × 70%). In this equation, the estimated blood volume was 75 mL/kg for males and 65 mL/kg for females, the mean volume of red blood cell was 213 mL, and the mean hematocrit was 70% [21, 22]. Intraoperative hypotension was defined as a mean arterial blood pressure < 65 mmHg for > 5 minutes recorded in the intraoperative anesthesia records. Postoperative outcomes included postoperative urinary infection, postoperative urinary obstruction, postoperative nonsteroidal anti-inflammatory drug (NSAID) use, hospital stay duration, AKI status, and ESRD status.

### 2.5. Statistical Analysis

Categorical variables are expressed as the number (%), and continuous variables are expressed as the mean ± standard deviation. Categorical variables were compared using the chi-square test or the Fisher's exact test, and continuous variables were compared using the Student's *t*-test or the Mann–Whitney *U*-test between the AKI and non-AKI groups. Univariate and multivariate logistic regression analyses were performed to identify independent risk factors for AKI. The most relevant factors associated with AKI were included in the univariate logistic regression analysis. Multivariate logistic regression analysis using the backward conditional method included all covariates with *P* < 0.05 from the univariate logistic regression analysis. The ability of the preoperative CRP/albumin ratio for predicting AKI after radical cystectomy was determined by calculating the area under the receiver operating characteristic (ROC) curve using the trapezoid rule. The optimal cut-off value was determined by the maximum value of sensitivity and specificity. The variance inflation factor was examined to assess multicollinearity. The Hosmer–Lemeshow goodness-of-fit statistic and the C-statistic were used to measure the calibration and discrimination of the logistic regression model.

To elucidate the impact of the CRP/albumin ratio on postoperative AKI among elderly patients, a 1 : 1 propensity score-matched analysis was conducted using the nearest-neighbor method with a 0.2 caliper size. The propensity score was calculated using logistic regression analysis to reduce selection bias and confounding factors. The standardized mean difference (SMD) was measured to estimate the balance between the two groups before and after propensity score matching. After 1 : 1 propensity score matching, categorical variables were compared using McNemar's test, and continuous variables were compared using the paired *t*-test. Conditional logistic regression analysis was conducted to evaluate the ability of the CRP/albumin ratio to predict the incidence of postoperative AKI in the propensity score-matched cohort. All statistical analyses were carried out using SPSS Statistics for Windows, version 21.0 (IBM Corp., Armonk, NY, USA), and Stata, version 13.1 (Stata Corp., College Station, TX, USA). *P* values < 0.05 were considered statistically significant.

## 3. Results

**Figure 1:**
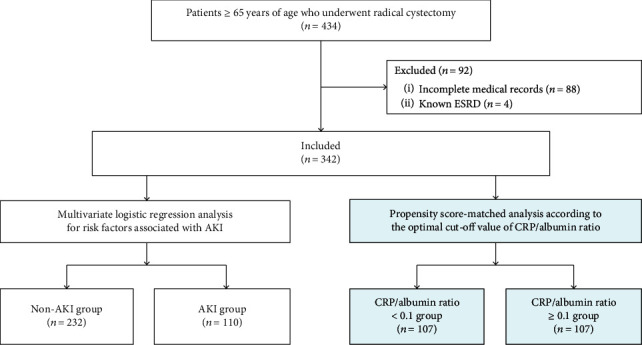
Flowchart of study patient enrollment: 434 elderly patients who underwent radical cystectomy were evaluated, and 342 elderly patients were included in the study. Patients were categorized according to the occurrence of AKI, and multivariate logistic regression analysis was performed. Then, patients were dichotomized according to the optimal CRP/albumin ratio cut-off value (i.e., 0.1), and a propensity score-matched analysis was performed. ESRD: end stage renal disease; AKI: acute kidney injury; CRP: C-reactive protein.

Among 434 elderly patients who underwent radical cystectomy between August 2007 and December 2018, 92 patients were excluded because of incomplete medical records and known ESRD. Therefore, 342 elderly patients were included in the analysis (Figure 1). One hundred ten patients (32.2%) had AKI after radical cystectomy (stage 1, 91/110; stage 2, 17/110; and stage 3, 2/110). None of the AKI patients had urinary tract obstruction during their hospital stays, 58 used perioperative NSAIDs, and 16 had intraoperative hypotension.

**Figure 2:**
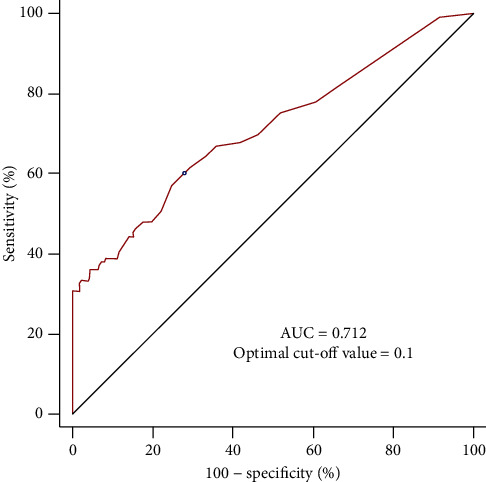
Receiver operating characteristic curve analysis of the CRP/albumin ratio to predict AKI after radical cystectomy. The AUC is 0.712, with an optimal cut-off value of 0.1. AUC: area under the curve; CRP: C-reactive protein; AKI: acute kidney injury.

Patient demographic and clinical characteristics, as well as intraoperative and postoperative data, are listed in Tables 1 and 2. Multivariate logistic regression analysis showed that the CRP/albumin ratio (odds ratio (OR) = 21.747, 95% confidence interval (CI) = 6.506–72.683, *P* < 0.001) and 6% hydroxyethyl starch amount (OR = 1.053, 95% CI = 1.014–1.093, *P* = 0.007) were significantly associated with AKI after radical cystectomy (Table 3). The ROC curve analysis revealed that the area under the curve of the CRP/albumin ratio was 0.712 and the optimal cut-off value was 0.1, with a sensitivity of 61.8% and specificity of 72.4% (Figure 2). The all variance inflation factor was < 10, ensuring a lack of multicollinearity. The Hosmer–Lemeshow goodness-of-fit probability was 0.413, and the C-statistic for the model was 0.742, with good calibration and discrimination.

Patients were dichotomized according to the optimal CRP/albumin ratio cut-off value (i.e., 0.1), as determined by the ROC curve analysis. Of the 342 patients, 206 (60.2%) had a CRP/albumin ratio < 0.1, and 136 (39.8%) had a CRP/albumin ratio ≥ 0.1 (Table 4). After applying 1 : 1 propensity score matching, 107 matched pairs were generated, and the patients were divided into a CRP/albumin ratio < 0.1 group (*n* = 107) and a CRP/albumin ratio ≥ 0.1 group (*n* = 107) (Table 4). All covariates were well-balanced, with an SMD < 0.2, and there were no significant differences between the two groups (Table 4). After applying 1 : 1 propensity score matching, the intraoperative data and postoperative outcomes of the patients, including operation time, crystalloid and 6% hydroxyethyl starch amounts, estimated blood loss, intraoperative hypotension, postoperative urinary infection, postoperative urinary tract obstruction, postoperative NSAID use, and hospital stay duration were not significantly different between the two groups (Table 5).

**Figure 3:**
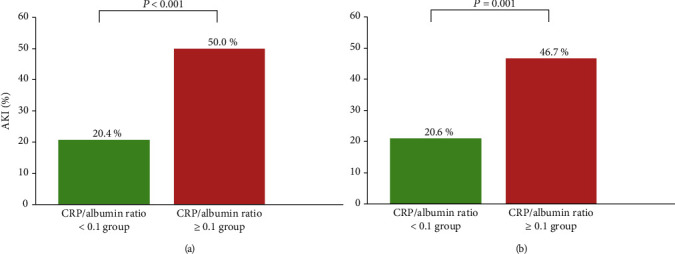
Comparison of the incidences of AKI after radical cystectomy between the CRP/albumin ratio ≥ 0.1 and CRP/albumin ratio < 0.1 groups before (a) and after (b) propensity score matching. The incidences of AKI in the CRP/albumin ratio ≥ 0.1 group were significantly higher than those in the CRP/albumin ratio < 0.1 group both before and after propensity score matching. AKI: acute kidney injury; CRP: C-reactive protein.

**Figure 4:**
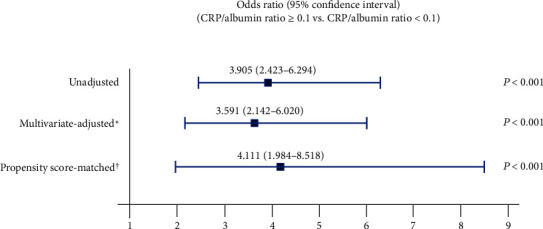
Predictive ability of a CRP/albumin ratio ≥ 0.1 for the occurrence of acute kidney injury after radical cystectomy among elderly patients. ^∗^The multivariate-adjusted odds ratio was adjusted using the variables in Table 3. ^†^The propensity score matching was performed using the variables in Table 4. CRP: C-reactive protein.

The incidences of AKI in the CRP/albumin ratio ≥ 0.1 group were significantly higher than those in the CRP/albumin ratio < 0.1 group both before (50.0% [68/136] vs. 20.4% [42/206], *P* < 0.001) and after (46.7% [50/107] vs. 20.6% [22/107], *P* < 0.001) propensity score matching (Figure 3). The ability of a CRP/albumin ratio ≥ 0.1 to predict the incidence of AKI after radical cystectomy among elderly patients is summarized in Figure 4. The incidences of AKI in the CRP/albumin ratio ≥ 0.1 group were significantly higher than those in the CRP/albumin ratio < 0.1 group in the unadjusted (OR = 3.905, 95% CI = 2.423–6.294, *P* < 0.001), multivariate-adjusted (OR = 3.591, 95% CI = 2.142–6.020, *P* < 0.001), and propensity score-matched cohorts (OR = 4.111, 95% CI = 1.984–8.518, *P* < 0.001) (Figure 4).

**Figure 5:**
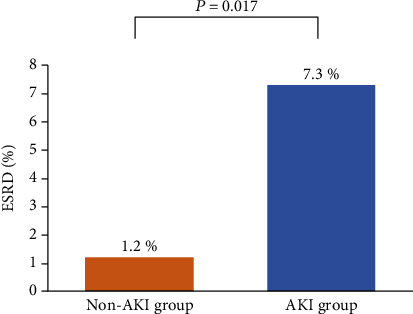
Comparison of the incidence of ESRD between the non-AKI and AKI groups after radical cystectomy among elderly patients. ESRD: end-stage renal disease; AKI: acute kidney injury.

ESRD was evaluated in 249 elderly patients at 12 months after radical cystectomy. Patients with incomplete medical records (*n* = 93) were excluded. The incidence of ESRD was significantly higher in the AKI group than in the non-AKI group at this time point (7.3% [6/82] vs. 1.2% [2/167], *P* = 0.017) (Figure 5). The CRP/albumin ratio was significantly higher among patients with ESRD than among those without ESRD at 12 months after radical cystectomy (0.59 ± 0.86 vs. 0.19 ± 0.44, *P* = 0.015).

## 4. Discussion

We observed that AKI after radical cystectomy occurred in 32.2% of the elderly patients included in this study. The CRP/albumin ratio and intraoperative 6% hydroxyethyl starch amount were independent risk factors associated with AKI after radical cystectomy. The optimal preoperative CRP/albumin ratio cut-off value for predicting AKI in this study was 0.1. After propensity score matching, the incidence of AKI in the CRP/albumin ratio ≥ 0.1 group was significantly higher than that in the CRP/albumin ratio < 0.1 group, and a preoperative CRP/albumin ratio ≥ 0.1 was significantly associated with an increased incidence of AKI after radical cystectomy among elderly patients. ESRD at 12 months after radical cystectomy occurred more frequently in the AKI group than in the non-AKI group.

Radical cystectomy is among the most complex and high-risk surgical procedures in urology [23, 24]. Elderly patients are more vulnerable to postoperative complications and poor outcomes associated with radical cystectomy [25]. In particular, postoperative AKI is among the most common complications associated with radical cystectomy. In the present study, the incidence of AKI after radical cystectomy was 32.2% among elderly patients, while the incidence of AKI after major abdominal surgery has been reported to be approximately 13% in the general population [26]. With aging, the kidneys undergo anatomic changes, including decreased renal mass, glomerulosclerosis, decreased tubular size and number, and tubulointerstitial fibrosis. The kidneys also undergo age-related physiologic changes, including decreased renal blood flow and glomerular filtration rate, diminished urinary concentrating and diluting ability, and decreased plasma renin and aldosterone levels. These changes result in renal functional reserve loss and increased sensitivity to pathological stresses and medications, leading to increased susceptibility to AKI [27]. Moreover, among elderly patients, serum creatinine elevations following AKI might be underestimated due to reduced muscle mass [27]. Therefore, meticulous perioperative management and risk assessments are required to reduce or prevent the occurrence of AKI after radical cystectomy in elderly patients.

In the present study, the CRP/albumin ratio was significantly higher in the AKI group. AKI is associated with inflammatory status [28]. Several studies have demonstrated that CRP is significantly associated with AKI [29, 30]. CRP is considered a useful tool for detecting active inflammatory processes. CRP is a powerful chemoattractant that promotes the expression of adhesion molecules, potentiates plasminogen activator inhibitor-1, and diminishes nitric oxide production. Increased CRP levels result in endothelial dysfunction, which induces vasoconstrictive, prothrombotic, and proinflammatory pathways [31]. Then, the platelet and coagulation systems are activated, leading to reduced renal blood flow and oxygen delivery [32]. Therefore, elevated CRP levels could influence the occurrence of AKI. Furthermore, serum albumin levels reflect a patient's nutritional and inflammatory status. The synthesis of serum albumin is inhibited by malnutrition and inflammation. Serum albumin is reported to have renoprotective effects; it acts to prolong potent renal vasodilation, which improves glomerular filtration and renal perfusion [33]. Additionally, serum albumin suppresses apoptosis of renal tubular cells by discarding reactive oxygen species and carrying protective lysophosphatidic acid; it also proliferates renal tubular cells by activating phosphatidylinositol 3-kinase [34, 35]. Therefore, elevated CRP and decreased albumin levels might contribute to the occurrence of AKI after radical cystectomy among elderly patients. Several studies have shown that the CRP/albumin ratio has a prognostic value for diseases wherein inflammation plays an important role [11, 36, 37]. A previous study evaluating the effect of the CRP/albumin ratio on overall survival after radical cystectomy in bladder cancer patients found that a high CRP/albumin ratio was the most effective prognostic indicator [36]. In another study, the association between the CRP/albumin ratio and therapy responsiveness was assessed among patients with severe acute ulcerative colitis. It was found that the CRP/albumin ratio was a more accurate predictor of therapy responsiveness than CRP or albumin alone [37]. Additionally, the CRP/albumin ratio has been demonstrated to have a prognostic value, in terms of overall survival, comparable to other inflammation-based prognostic scores, such as the Glasgow Prognostic Score, the modified Glasgow Prognostic Score, and the neutrophil/lymphocyte ratio, for patients with hepatocellular carcinoma [11, 36, 37].

Aging is characterized by decreased immune function and physiological deterioration [38–40], and elderly people are often in a chronic inflammatory state, which is characterized by chronic heightened levels of inflammatory biomarkers [38]. Several previous studies have reported a chronic low-grade inflammatory status in elderly patients; this has been termed “inflamm-aging” [39, 40]. Circulating inflammatory mediators are associated with cardiovascular disease, sarcopenia, atherosclerosis, and mortality and may be sensitive markers of subclinical disorders in elderly patients [39]. The CRP/albumin ratio, which is an inflammation-based prognostic factor, has been reported to be higher among elderly patients than younger patients. Turco et al. demonstrated that elderly patients with ST-segment elevation myocardial infarction undergoing percutaneous coronary intervention had higher CRP/albumin ratios than younger patients [41]. The unfavorable inflammatory status among elderly patients can negatively affect postoperative renal function. Therefore, for elderly patients with chronic low-grade inflammation, the CRP/albumin ratio might be an important biomarker of AKI after radical cystectomy.

The present study also demonstrated that the intraoperative 6% hydroxyethyl starch amount was associated with postoperative AKI among elderly patients who underwent radical cystectomy. The issue of renal function after the intraoperative use of synthetic colloids remains controversial. Our findings agree with previous studies that have suggested that colloids might be associated with kidney dysfunction [42, 43]. The proposed mechanism of AKI development by synthetic colloid solutions is osmotic nephrosis. Synthetic colloid solutions cause hyperviscosity of the urine, which results in the obstruction of the tubular lumen of the kidney, leading to tubular nephrosis, resulting in renal toxicity [42]. However, inconsistent with our study, several trials have demonstrated that synthetic colloids are as safe as crystalloids for intraoperative use, with no evidence of an association with renal insufficiency [44]. Furthermore, a recent study showed that synthetic colloid-based intraoperative goal-directed fluid therapy was associated with a significantly lower incidence of postoperative complications, including renal failure [45]. Nonetheless, the intraoperative use of synthetic colloid solutions is not recommended for patients at risk of AKI [46, 47]. Therefore, clinicians should be cautious when considering the use of synthetic colloids to improve postoperative outcomes for elderly radical cystectomy patients.

It is remarkable that we demonstrated a negative impact of postoperative AKI on ESRD at 12 months after radical cystectomy. AKI has been considered to be self-limiting, with a good prognosis when recovery from AKI occurs during hospitalization [48]. However, postoperative AKI was associated with a high incidence of ESRD in our present cohort. In concordance with our study, AKI has been shown to be associated with a high risk of ESRD in the elderly [49]. Furthermore, the CRP/albumin ratio was significantly higher among patients with ESRD than among those without ESRD at 12 months after radical cystectomy in our study. These results suggest that the preoperative CRP/albumin ratio can predict both short-term and long-term changes in postoperative renal function. Therefore, preoperative evaluation of the CRP/albumin ratio can provide useful information regarding the occurrence of AKI and ESRD after radical cystectomy among elderly patients.

This study had several limitations. First, this study had biases associated with its retrospective design. However, we tried to include all possible factors potentially associated with AKI after radical cystectomy and performed a propensity score-matched analysis to minimize the bias. Second, because our study included patients who underwent radical cystectomy performed by highly specialized surgeons at a single large center, our results may not be generalizable and should be interpreted cautiously. Third, since 12.6% (43/342) patients died during the 12 months after radical cystectomy, the death event could be a bias in the evaluation of ESRD.

## 5. Conclusion

AKI occurred in 32.2% of elderly patients after radical cystectomy. The CRP/albumin ratio was an independent risk factor for postoperative AKI. A CRP/albumin ratio ≥ 0.1 was significantly associated with an increased incidence of AKI after radical cystectomy among elderly patients. Moreover, ESRD incidence at 12 months after radical cystectomy was significantly higher among elderly patients who had postoperative AKI. Therefore, elderly patients with elevated preoperative CRP/albumin ratios should be carefully managed to minimize or prevent postoperative AKI and subsequent ESRD after radical cystectomy.

## Figures and Tables

**Table 1:** Demographic and clinical characteristics of elderly cystectomy patients.

Variables	All patients (*n* = 342)	Non-AKI group (*n* = 232)	AKI group (*n* = 110)	*P* value^∗^
Sex (male)	280 (81.9)	184 (79.3)	96 (87.3)	0.098
Age (years)	72.1 ± 4.9	71.9 ± 4.9	72.6 ± 4.7	0.221
Body mass index (kg/m^2^)	23.9 ± 3.1	23.9 ± 3.0	23.9 ± 3.3	0.975
ASA physical status				0.868
≤2	294 (86.0)	200 (86.2)	94 (85.5)	
3	48 (14.0)	32 (13.8)	16 (14.5)	
Diabetes mellitus	79 (23.1)	56 (24.1)	23 (20.9)	0.583
Hypertension	175 (51.2)	125 (53.9)	50 (45.5)	0.165
Coronary artery disease	27 (7.9)	15 (6.5)	12 (10.9)	0.197
Cerebrovascular disease	16 (4.7)	13 (5.6)	3 (2.7)	0.286
COPD	17 (5.0)	10 (4.3)	7 (6.4)	0.431
Smoking history				0.876
Nonsmoker	169 (49.4)	117 (50.4)	52 (47.3)	
Current smoker	26 (7.6)	17 (7.3)	9 (8.2)	
Ex-smoker	147 (43.0)	98 (42.2)	49 (44.5)	
Tumor stage				0.767
1	34 (9.9)	22 (9.5)	12 (10.9)	
2	197 (57.6)	138 (59.5)	59 (53.6)	
3	69 (20.2)	44 (19.0)	25 (22.7)	
4	42 (12.3)	28 (12.1)	14 (12.7)	
Tumor grade				>0.999
2	9 (2.6)	6 (2.6)	3 (2.7)	
3	333 (97.4)	226 (97.4)	107 (97.3)	
Neoadjuvant chemotherapy	78 (22.8)	59 (25.4)	19 (17.3)	0.100
Preoperative laboratory tests				
White blood cells (10^3^/*μ*L)	6.8 ± 2.7	6.6 ± 2.2	7.2 ± 3.4	0.037
Hemoglobin (g/dL)	11.9 ± 1.9	12.2 ± 1.9	11.4 ± 1.9	<0.001
Platelets (10^3^/*μ*L)	241.9 ± 84.2	245.8 ± 81.4	235.6 ± 89.8	0.209
Albumin (g/dL)	3.6 ± 0.5	3.7 ± 0.4	3.4 ± 5.5	0.001
Creatinine (mg/dL)	1.0 ± 0.4	1.0 ± 0.3	1.1 ± 0.4	0.032
eGFR (mL/min/1.73m^2^)	72.7 ± 15.6	73.6 ± 15.9	70.9 ± 15.0	0.134
Sodium (mmol/L)	139.4 ± 3.1	139.6 ± 2.7	139.0 ± 3.8	0.099
Potassium (mmol/L)	4.4 ± 0.4	4.4 ± 0.4	4.4 ± 0.4	0.346
Chloride (mmol/L)	104.4 ± 3.5	104.4 ± 3.1	104.4 ± 4.2	0.959
Uric acid (mg/dL)	5.1 ± 1.5	5.1 ± 1.5	5.2 ± 1.6	0.838
CRP (mg/dL)	1.0 ± 2.2	0.4 ± 0.4	2.2 ± 3.4	<0.001
CRP/albumin ratio	0.33 ± 0.86	0.10 ± 0.13	0.79 ± 1.40	<0.001

Continuous variables are presented as the mean ± standard deviation and categorical variables as number (%). ^∗^For comparisons between the AKI and non-AKI groups. AKI: acute kidney injury; ASA: American Society of Anesthesiologists; COPD: chronic obstructive pulmonary disease; eGFR: estimated glomerular filtration rate; CRP: C-reactive protein.

**Table 2:** Intraoperative and postoperative data of elderly cystectomy patients.

Variables	All patients (*n* = 342)	Non-AKI group (*n* = 232)	AKI group (*n* = 110)	*P* value^∗^
Operation duration (min)	413.1 ± 110.1	409.0 ± 109.2	421.8 ± 111.9	0.314
Anesthesia duration (min)	437.2 ± 101.6	434.7 ± 99.8	442.3 ± 105.7	0.517
Crystalloid amount (mL/kg)	50.3 ± 22.3	50.1 ± 22.2	50.7 ± 22.7	0.827
Colloid amount				
6% hydroxyethyl starch (mL/kg)	7.4 ± 7.0	6.6 ± 6.5	9.2 ± 7.8	0.001
5% albumin (mL)	32.2 ± 88.1	34.5 ± 92.4	27.3 ± 78.3	0.480
Red blood cell transfusion	196 (57.3)	120 (51.7)	76 (69.1)	0.003
Estimated blood loss (mL)	899.9 ± 300.4	891.9 ± 289.4	916.7 ± 323.0	0.476
Intraoperative hypotension	46 (13.5)	30 (12.9)	16 (14.5)	0.735
NMB reversal agent				0.646
Neostigmine-glycopyrrolate	283 (82.7)	190 (81.9)	93 (84.5)	
Sugammadex	59 (17.3)	42 (18.1)	17 (15.5)	
Urinary diversion type				0.015
Ileal conduit	181 (52.9)	112 (48.3)	69 (62.7)	
Ileal neobladder	161 (47.1)	120 (51.7)	41 (37.3)	
Postoperative urinary infection	77 (22.5)	25 (22.7)	52 (22.4)	>0.999
Postoperative urinary tract obstruction	1 (0.3)	0 (0.0)	1 (0.4)	>0.999
Postoperative NSAID use	160 (46.8)	58 (52.7)	102 (44.0)	0.134

Continuous variables are presented as the mean ± standard deviation and categorical variables as number (%). ^∗^For comparisons between the AKI and non-AKI groups. AKI: acute kidney injury; NMB: neuromuscular blockade; NSAID: nonsteroidal anti-inflammatory drug.

**Table 3:** Univariate and multivariate logistic regression analyses of risk factors for acute kidney injury after radical cystectomy among elderly patients.

Variables	Univariate analysis		Multivariate analysis	
	OR (95% CI)	*P* value	OR (95% CI)	*P* value
Sex (female)	0.559 (0.293–1.065)	0.077		
Age	1.029 (0.983–1.078)	0.221		
Body mass index	1.001 (0.931–1.077)	0.975		
ASA physical status				
≤2	1.000			
3	1.064 (0.556–2.034)	0.852		
Diabetes mellitus	0.831 (0.480–1.439)	0.508		
Hypertension	0.713 (0.452–1.125)	0.146		
Coronary artery disease	1.771 (0.799–3.925)	0.159		
Cerebrovascular accident	0.472 (0.132–1.693)	0.249		
COPD	1.509 (0.559–4.076)	0.417		
Tumor stage				
1	1.000			
2	0.784 (0.364–1.687)	0.533		
3	1.042 (0.442–2.456)	0.926		
4	0.917 (0.354–2.375)	0.858		
Tumor grade				
2	1.000			
3	0.947 (0.232–3.859)	0.939		
Neoadjuvant chemotherapy	0.612 (0.344–1.089)	0.095		
White blood cells	1.091 (1.003–1.186)	0.042		
Hemoglobin	0.804 (0.710–0.910)	0.001		
eGFR	0.989 (0.975–1.003)	0.135		
Uric acid	1.016 (0.874–1.181)	0.837		
CRP/albumin ratio	20.398 (6.379–65.227)	<0.001	21.747 (6.506–72.683)	<0.001
Operation duration	1.001 (0.999–1.003)	0.313		
Crystalloid amount	1.001 (0.991–1.011)	0.826		
6% hydroxyethyl starch amount	1.054 (1.020–1.089)	0.002	1.053 (1.014–1.093)	0.007
Red blood cell transfusion	2.086 (1.292–3.370)	0.003		
Estimated blood loss	1.000 (1.000–1.001)	0.475		
Intraoperative hypotension	1.146 (0.596–2.205)	0.683		
Urinary diversion type				
Ileal conduit	1.000			
Ileal neobladder	0.555 (0.349–0.882)	0.013		
Postoperative urinary infection	1.018 (0.592–1.751)	0.948		
Postoperative urinary tract obstruction	0.000 (0.000–0.000)	>0.999		
Postoperative NSAID use	1.422 (0.902–2.241)	0.130		

OR: odds ratio; CI: confidence interval; ASA: American Society of Anesthesiologists; COPD: chronic obstructive pulmonary disease; eGFR: estimated glomerular filtration rate; CRP: C-reactive protein; NSAID: nonsteroidal anti-inflammatory drug. Multivariate logistic regression analysis included white blood cells, hemoglobin, CRP/albumin ratio, 6% hydroxyethyl starch amount, red blood cell transfusion, and urinary diversion type from the univariate logistic regression analysis.

**Table 4:** Demographic and clinical characteristics of elderly cystectomy patients dichotomized according to the optimal CRP/albumin ratio cut-off value (0.1) before and after propensity score matching.

Variable	Before propensity score matching				After propensity score matching			
	CRP/albumin ratio <0.1 group (*n* = 206)	CRP/albumin ratio ≥0.1 group (*n* = 136)	SMD	*P* value	CRP/albumin ratio <0.1 group (*n* = 107)	CRP/albumin ratio ≥0.1 group (*n* = 107)	SMD	*P* value
Sex (male)	171 (83.0)	109 (80.1)	-0.072	0.567	85 (79.4)	86 (80.4)	0.023	>0.999
Age (years)	71.5 ± 4.7	73.0 ± 5.0	0.297	0.005	72.5 ± 5.1	72.4 ± 4.8	-0.011	0.932
Body mass index (kg/m^2^)	24.3 ± 2.8	23.4 ± 3.5	-0.263	0.008	23.9 ± 3.0	23.6 ± 3.4	-0.107	0.352
ASA physical status			0.096	0.427			0.126	0.405
≤2	180 (87.4)	114 (83.8)			5 (88.8)	90 (84.1)		
3	26 (12.6)	22 (16.2)			12 (11.2)	17 (15.9)		
Diabetes mellitus	53 (25.7)	26 (19.1)	-0.167	0.190	23 (21.5)	21 (19.6)	-0.047	0.851
Hypertension	108 (52.4)	67 (49.3)	-0.063	0.582	55 (51.4)	56 (52.3)	0.019	>0.999
Coronary artery disease	13 (6.3)	14 (10.3)	0.131	0.220	8 (7.5)	10 (9.3)	0.061	0.804
Cerebrovascular disease	9 (4.4)	7 (5.1)	0.035	0.796	3 (2.8)	6 (5.6)	0.126	0.508
COPD	10 (4.9)	7 (5.1)	0.013	>0.999	7 (6.5)	5 (4.7)	-0.084	0.774
Tumor stage			-0.056	0.639			-0.020	>0.999
<3	137 (66.5)	94 (69.1)			74 (69.2)	75 (70.1)		
≥3	69 (33.5)	42 (30.9)			33 (30.8)	32 (29.9)		
Tumor grade			0.048	0.747			0.127	0.625
2	6 (2.9)	3 (2.2)			3 (2.8)	1 (0.9)		
3	200 (97.1)	133 (97.8)			104 (97.2)	106 (99.1)		
Neoadjuvant chemotherapy	44 (21.4)	34 (25.0)	0.084	0.433	26 (24.3)	27 (25.2)	0.022	>0.999
White blood cells (10^3^/*μ*L)	6.351 ± 2.021	7.424 ± 3.327	0.322	<0.001	6.610 ± 2.121	6.560 ± 1.948	-0.015	0.856
Hemoglobin (g/dL)	12.4 ± 1.8	11.2 ± 1.9	-0.601	<0.001	11.7 ± 1.8	11.6 ± 1.8	-0.046	0.645
eGFR (mL/min/1.73m^2^)	74.3 ± 14.1	70.2 ± 17.5	-0.236	0.017	71.8 ± 14.4	71.9 ± 16.5	0.005	0.966
Uric acid (mg/dL)	5.1 ± 1.4	5.2 ± 1.7	0.099	0.327	5.1 ± 1.4	5.2 ± 1.7	0.061	0.660

Continuous variables are presented as the mean ± standard deviation and categorical variables as number (%). CRP: C-reactive protein; SMD: standardized mean difference; ASA: American Society of Anesthesiologists; COPD: chronic obstructive pulmonary disease; eGFR: estimated glomerular filtration rate.

**Table 5:** Intraoperative data and postoperative outcomes of elderly cystectomy patients dichotomized according to the optimal CRP/albumin ratio cut-off value (0.1) after propensity score matching.

Variables	All patients (*n* = 214)	CRP/albumin ratio <0.1 group (*n* = 107)	CRP/albumin ratio ≥0.1 group (*n* = 107)	*P* value
Operation time (min)	410.0 ± 108.4	398.5 ± 109.4	421.4 ± 106.6	0.143
Crystalloid amount (mL)	3165.9 ± 1281.0	3062.6 ± 1326.8	3269.2 ± 1231.0	0.256
6% hydroxyethyl starch amount (mL)	442.3 ± 426.1	408.0 ± 410.4	476.6 ± 440.5	0.242
Estimated blood loss (mL)	881.9 ± 306.1	855.3 ± 308.7	908.5 ± 302.5	0.170
Intraoperative hypotension	33 (15.4)	20 (18.7)	13 (12.1)	0.296
Postoperative urinary infection	47 (22.0)	23 (21.5)	24 (22.4)	>0.999
Postoperative urinary tract obstruction	1 (0.5)	1 (0.9)	0 (0.0)	>0.999
Postoperative NSAID use	96 (44.9)	42 (39.3)	54 (50.5)	0.141
Hospital stay (days)	25.3 ± 25.9	23.4 ± 14.9	27.3 ± 33.5	0.267

Continuous variables are presented as the mean ± standard deviation and categorical variables as number (%). NSAID: nonsteroidal anti-inflammatory drug.

## Data Availability

The data used in the present study are available from the corresponding author upon reasonable request.

## References

[1] Babjuk M. (2018). Bladder cancer in the elderly. *European Urology*.

[2] Kim S. P., Boorjian S. A., Shah N. D. (2012). Contemporary trends of in-hospital complications and mortality for radical cystectomy. *BJU International*.

[3] Clark P. E., Stein J. P., Groshen S. G. (2005). Radical cystectomy in the elderly: comparison of clincal outcomes between younger and older patients. *Cancer*.

[4] Joung K. W., Kong Y. G., Yoon S. H. (2016). Comparison of postoperative acute kidney injury between ileal conduit and neobladder urinary diversions after radical cystectomy: a propensity score matching analysis. *Medicine*.

[5] Santacruz F., Barreto S., Mayor M. M., Cabrera W., Breuer N. (2009). Mortality in elderly patients with acute renal failure. *Renal Failure*.

[6] Coca S. G. (2010). Acute kidney injury in elderly persons. *American Journal of Kidney Diseases*.

[7] Morris-Stiff G., Gomez D., Prasad K. R. (2008). C-reactive protein in liver cancer surgery. *European Journal of Surgical Oncology*.

[8] Ticinesi A., Lauretani F., Nouvenne A. (2017). C-reactive protein (CRP) measurement in geriatric patients hospitalized for acute infection. *European Journal of Internal Medicine*.

[9] Murat S. N., Kurtul A., Yarlioglues M. (2014). Impact of serum albumin levels on contrast-induced acute kidney injury in patients with acute coronary syndromes treated with percutaneous coronary intervention. *Angiology*.

[10] Don B. R., Kaysen G. (2004). Serum albumin: relationship to inflammation and nutrition. *Seminars in Dialysis*.

[11] Kinoshita A., Onoda H., Imai N. (2015). The C-reactive protein/albumin ratio, a novel inflammation-based prognostic score, predicts outcomes in patients with hepatocellular carcinoma. *Annals of Surgical Oncology*.

[12] Fairclough E., Cairns E., Hamilton J., Kelly C. (2009). Evaluation of a modified early warning system for acute medical admissions and comparison with C-reactive protein/albumin ratio as a predictor of patient outcome. *Clinical Medicine*.

[13] Yu J., Hong B., Park J.-Y., Hwang J.-H., Kim Y.-K. (2020). Impact of prognostic nutritional index on postoperative pulmonary complications in radical cystectomy: a propensity score-matched analysis. *Annals of Surgical Oncology*.

[14] Jeong I. G., You D., Kim J. W. (2011). Outcomes of single lymph node positive urothelial carcinoma after radical cystectomy. *Journal of Urology*.

[15] Yu J., Hong B., Park J.-Y. (2020). Comparison of a significant decline in the glomerular filtration rate between ileal conduit and ileal neobladder urinary diversions after radical cystectomy: a propensity score-matched analysis. *Journal of Clinical Medicine*.

[16] Okusa M. D., Davenport A. (2014). Reading between the (guide)lines: the KDIGO practice guideline on acute kidney injury in the individual patient. *Kidney International*.

[17] Lane B. R., Babineau D. C., Poggio E. D. (2008). Factors predicting renal functional outcome after partial nephrectomy. *The Journal of Urology*.

[18] Levey A. S., Stevens L. A., Schmid C. H. (2009). A new equation to estimate glomerular filtration rate. *Annals of Internal Medicine*.

[19] Edge S. B., Compton C. C. (2010). The American Joint Committee on Cancer: the 7th edition of the *AJCC cancer staging manual* and the future of TNM. *Annals of Surgical Oncology*.

[20] Comperat E. M., Burger M., Gontero P. (2019). Grading of urothelial carcinoma and the new "World Health Organisation Classification of Tumours of the Urinary System and Male Genital Organs 2016". *European Urology Focus*.

[21] Furman E. B., Roman D. G., Lemmer J. H., Jasinska M., Laver M. B. (1975). Specific therapy in water, electrolyte and blood-volume replacement during pediatric surgery. *Anesthesiology*.

[22] Bang S. R., Ahn H. J., Kim G. S. (2010). Predictors of high intraoperative blood loss derived by simple and objective method in adult living donor liver transplantation. *Transplantation Proceedings*.

[23] Chang S. S., Cookson M. S., Baumgartner R. G., Wells N., Smith J. A. (2002). Analysis of early complications after radical cystectomy: results of a collaborative care pathway. *Journal of Urology*.

[24] Shabsigh A., Korets R., Vora K. C. (2009). Defining early morbidity of radical cystectomy for patients with bladder cancer using a standardized reporting methodology. *European Urology*.

[25] Prout G. R., Wesley M. N., Yancik R., Ries L. A., Havlik R. J., Edwards B. K. (2005). Age and comorbidity impact surgical therapy in older bladder carcinoma patients. *Cancer*.

[26] O’Connor M. E., Kirwan C. J., Pearse R. M., Prowle J. R. (2016). Incidence and associations of acute kidney injury after major abdominal surgery. *Intensive Care Medicine*.

[27] Abdel-Kader K., Palevsky P. M. (2009). Acute kidney injury in the elderly. *Clinics in Geriatric Medicine*.

[28] Rabb H., Griffin M. D., McKay D. B. (2016). Inflammation in AKI: current understanding, key questions, and knowledge gaps. *Journal of the American Society of Nephrology*.

[29] Karabag Y., Cagdas M., Rencuzogullari I. (2019). The C-reactive protein to albumin ratio predicts acute kidney injury in patients with ST-segment elevation myocardial infarction undergoing primary percutaneous coronary intervention. *Heart, Lung & Circulation*.

[30] Lai W., Tang Y., Huang X. R. (2016). C-reactive protein promotes acute kidney injury via Smad3-dependent inhibition of CDK2/cyclin E. *Kidney International*.

[31] Yudkin J. S., Stehouwer C. D. A., Emeis J. J., Coppack S. W. (1999). C-reactive protein in healthy subjects: associations with obesity, insulin resistance, and endothelial dysfunction. *Arteriosclerosis, Thrombosis, and Vascular Biology*.

[32] Bisoendial R. J., Kastelein J. J. P., Levels J. H. M. (2005). Activation of inflammation and coagulation after infusion of C-reactive protein in humans. *Circulation Research*.

[33] Kaufmann M. A., Castelli I., Pargger H., Drop L. J. (1995). Nitric oxide dose-response study in the isolated perfused rat kidney after inhibition of endothelium-derived relaxing factor synthesis: the role of serum albumin. *The Journal of Pharmacology and Experimental Therapeutics*.

[34] Iglesias J., Abernethy V. E., Wang Z., Lieberthal W., Koh J. S., Levine J. S. (1999). Albumin is a major serum survival factor for renal tubular cells and macrophages through scavenging of ROS. *The American Journal of Physiology*.

[35] Dixon R., Brunskill N. J. (1999). Activation of mitogenic pathways by albumin in kidney proximal tubule epithelial cells: implications for the pathophysiology of proteinuric states. *Journal of the American Society of Nephrology*.

[36] Guo Y., Cai K., Mao S. (2018). Preoperative C-reactive protein/albumin ratio is a significant predictor of survival in bladder cancer patients after radical cystectomy: a retrospective study. *Cancer Management and Research*.

[37] Gibson D. J., Hartery K., Doherty J. (2018). CRP/albumin ratio: an early predictor of steroid responsiveness in acute severe ulcerative colitis. *Journal of Clinical Gastroenterology*.

[38] Li H., Manwani B., Leng S. X. (2011). Frailty, inflammation, and immunity. *Aging and Disease*.

[39] Krabbe K. S., Pedersen M., Bruunsgaard H. (2004). Inflammatory mediators in the elderly. *Experimental Gerontology*.

[40] Ferrucci L., Corsi A., Lauretani F. (2005). The origins of age-related proinflammatory state. *Blood*.

[41] Del Turco S., Basta G., De Caterina A. R. (2019). Different inflammatory profile in young and elderly STEMI patients undergoing primary percutaneous coronary intervention (PPCI): its influence on no-reflow and mortality. *International Journal of Cardiology*.

[42] Rioux J. P., Lessard M., De Bortoli B. (2009). Pentastarch 10% (250 kDa/0.45) is an independent risk factor of acute kidney injury following cardiac surgery. *Critical Care Medicine*.

[43] Wiedermann C. J., Dunzendorfer S., Gaioni L. U., Zaraca F., Joannidis M. (2010). Hyperoncotic colloids and acute kidney injury: a meta-analysis of randomized trials. *Critical Care*.

[44] Marx G., Schindler A. W., Mosch C. (2016). Intravascular volume therapy in adults: guidelines from the Association of the Scientific Medical Societies in Germany. *European Journal of Anaesthesiology*.

[45] Joosten A., Delaporte A., Ickx B. (2018). Crystalloid versus colloid for intraoperative goal-directed fluid therapy using a closed-loop system: a randomized, double-blinded, controlled trial in major abdominal surgery. *Anesthesiology*.

[46] Hartog C. S., Natanson C., Sun J., Klein H. G., Reinhart K. (2014). Concerns over use of hydroxyethyl starch solutions. *BMJ*.

[47] Goren O., Matot I. (2015). Perioperative acute kidney injury. *British Journal of Anaesthesia*.

[48] Star R. A. (1998). Treatment of acute renal failure. *Kidney International*.

[49] Ishani A., Xue J. L., Himmelfarb J. (2009). Acute kidney injury increases risk of ESRD among elderly. *Journal of the American Society of Nephrology*.

